# Raman Method in Identification of Species and Varieties, Assessment of Plant Maturity and Crop Quality—A Review

**DOI:** 10.3390/molecules27144454

**Published:** 2022-07-12

**Authors:** Aneta Saletnik, Bogdan Saletnik, Czesław Puchalski

**Affiliations:** Department of Bioenergetics, Food Analysis and Microbiology, Institute of Food Technology and Nutrition, College of Natural Science, Rzeszow University, Ćwiklińskiej 2D, 35-601 Rzeszow, Poland; asaletnik@ur.edu.pl (A.S.); cpuchalski@ur.edu.pl (C.P.)

**Keywords:** Raman spectroscopy, digital farming, harvest maturity assessment, fruit and seeds quality diagnostics, non-invasive phenotyping

## Abstract

The present review covers reports discussing potential applications of the specificity of Raman techniques in the advancement of digital farming, in line with an assumption of yield maximisation with minimum environmental impact of agriculture. Raman is an optical spectroscopy method which can be used to perform immediate, label-free detection and quantification of key compounds without destroying the sample. The authors particularly focused on the reports discussing the use of Raman spectroscopy in monitoring the physiological status of plants, assessing crop maturity and quality, plant pathology and ripening, and identifying plant species and their varieties. In recent years, research reports have presented evidence confirming the effectiveness of Raman spectroscopy in identifying biotic and abiotic stresses in plants as well as in phenotyping and digital selection of plants in farming. Raman techniques used in precision agriculture can significantly improve capacities for farming management, crop quality assessment, as well as biological and chemical contaminant detection, thereby contributing to food safety as well as the productivity and profitability of agriculture. This review aims to increase the awareness of the growing potential of Raman spectroscopy in agriculture among plant breeders, geneticists, farmers and engineers.

## 1. Introduction

Population growth and consequently the increasing demand for food as well as the decreasing availability of fertile land have led to reduced agricultural expansion and higher costs of agriculture and food. All these factors, combined with the long history of conventional agriculture, have forced food producers to seek new solutions. Owing to advancements in technology, these problems can be solved by introducing innovative methods in agriculture. The transformation of farming processes with the use of smart technologies, referred to as digital agriculture, aims to develop innovative technological methods to be used to maximise yield and minimise the environmental impact of agriculture [1,2,3,4]. Digital agriculture requires advanced methodologies for cultivation and selection of plants [5,6], detection and identification of biotic and abiotic stresses in plants, and for the acquisition of information about the health of plants and growth stages directly on the plantation. These methods are highly needed by crop producers [7].

In the related literature, there is more and more evidence showing the effectiveness of Raman spectroscopy in identifying the physiological condition of plants, crop quality, plant species or varieties, in assessing the maturity of plants and in pre-symptomatic diagnostics, or in detecting abiotic and biotic stresses in plants [1,8,9,10,11,12,13,14,15,16,17]. Raman spectroscopy also enables phenotyping and digital selection of plants in breeding. Owing to the immediate access to information about plant health, it is possible to detect and identify bacterial infections, secondary diseases, insect infestations, fungal infections, and other pathogens and the diseases they transmit in greenhouses and fields [8,18,19]. The information acquired this way is used to perform precise and site-specific chemical treatments which may prevent the spread of biotic stresses and save up to 30% of the crop. Rapid detection of physiological drought or nutrient deficiencies allows for supply of the nutrients promptly and accurately. By applying fertilisers to a strictly defined area, it is possible to reduce contamination of the crops and the soil [20,21].

Moreover, a tool effectively assessing the ripeness of the fruit and the quality of the crop helps the farmer to make sure that the crop is harvested at the right time. Researchers have demonstrated in a number of studies that Raman spectroscopy is a perfect method for enabling monitoring of field and greenhouse crops because of its high selectivity and specificity [1]. Recent research findings show that Raman spectroscopy (RS) can effectively be used in diagnostics of biotic and abiotic stresses [22,23,24,25]. RS is a label-free laser technique that does not require chemicals to perform analysis of plant material, so the farmer does not incur any costs by purchasing reagents. Furthermore, it takes only one second to perform analysis of a plant in order to detect pathogens or to identify the origin of abiotic stresses [26].

Agriculture and crop production are relatively new areas in research involving Raman spectroscopy [8]. This review focuses particularly on RS application for evaluating the physiological status of plants as well as the stage in the ripening process, for assessing quality of crops (fruit and seeds), and for identifying plant species, varieties, and their origin. This review aims to increase the awareness of the potential of Raman spectroscopy in agriculture among plant breeders, geneticists, farmers, and engineers.

## 2. Principle, Instrumentation

Raman spectroscopy is used to examine the structure, dynamics of changes, and function of biomolecules. It is a vibrational technique providing insight into the structure of tissues and their components at the molecular level [27,28,29,30].

In principle, Raman spectroscopy measures the frequency shift in the inelastic scattering of light when a photon of incident light strikes a particle and produces a scattered photon [31,32,33,34,35,36]. In the light scattered by the test medium there is, for the most part, a component of the same frequency as in the incident light (Rayleigh scattering, elastic scattering) [36], while in a minority of cases there are variable frequency components associated with the change in photon energy (inelastic scattering, Raman scattering). The outgoing scattered light can be a photon with a frequency lower than the incident photon and in such cases we call it Stokes Raman scattering, or it is of a frequency that is higher, and then it is known as anti-Stokes Raman scattering [29]. The Stokes band forms when the molecule, after interacting with the excitation radiation, shifts to a higher oscillatory level and the scattered photon has energy lower than the energy difference between the levels of vibrational energy. On the other hand, the anti-Stokes band may appear if the molecule was at the excited oscillatory level before the impact of the excitation radiation – that way there is a high probability that it returns to the basic oscillatory level. The scattered photon has an energy greater than the difference in energy of the oscillating energy levels [36]. Since the Stokes bands are of higher intensity than the anti-Stokes bands, in Raman spectroscopy, the measurement most often concerns only the Stokes part of the Raman spectrum, presented in the range from 4000 to 0 Δ cm^−1^ (the so-called Raman shift) [29].

The phenomenon of Raman scattering of light by particles was predicted by Smekal in 1923 and observed experimentally for the first time by Sir Chandrasekhar Venkat Raman and his student Kariamanickam Srinivas Krishnan in 1928 [37,38].

**Figure 1:**
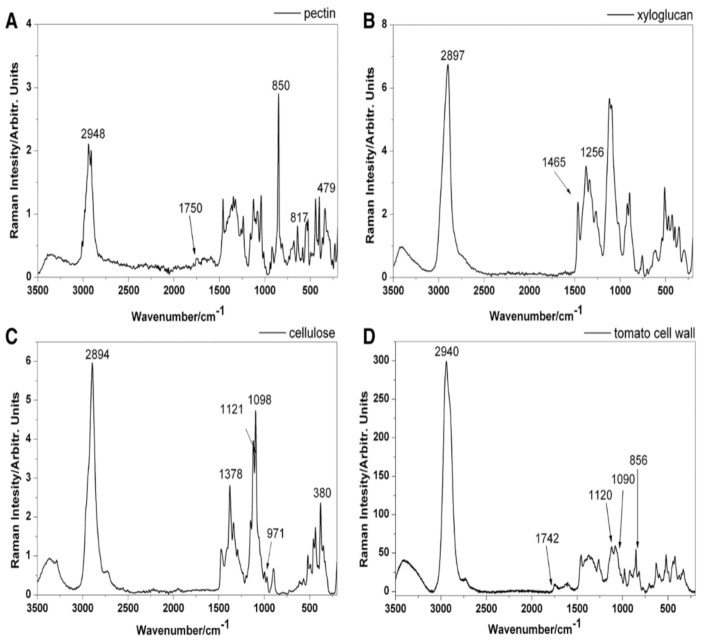
Raman spectra of the pure cell wall components (laser with green light at λ = 532 nm, with a power of 10 mW, the spectra were recorded within the range of 3500–150 cm−1): pectin (**A**), xyloglucan (**B**), cellulose (**C**), and the Raman spectrum of the tomato cell wall (**D**). Copyright Plant Methods 2014.

In honour of the discoverer, the phenomenon of inelastic scattering of light is called Raman scattering [39]. An advantage presented by Raman spectroscopy lies in the fact that when it is used to examine biological materials, the spectra containing large amounts of information can be acquired from intact tissues [40,41], without interfering in their structure. This way it is possible to perform detailed chemical analysis of the biological material despite its high complexity. Organic compounds and functional groups have characteristic spectral patterns, the so-called "fingerprints", enabling their identification, while the intensity of the bands can be used to calculate the concentration in the sample analysed [42]. The Raman spectrum can be used as a fingerprinting tool for various compounds [43]. Table 1 shows the assignment of bands in the Raman spectra of cell wall polysaccharides based on the literature, and Figure 1 shows the Raman spectra of the pure cell wall components pectin (A), xyloglucan (B), cellulose (C), and the Raman spectrum of the tomato cell wall (D) [44]. 

The obtained analyte spectrum can be treated as a qualitative analysis of unknown samples or mixtures of components [45]. Importantly, Raman spectroscopy is sensitive even to small structural changes, which is useful in comparative studies [41]. Raman scattering in tissues provides a wealth of information about the vibrational structure of their constituent proteins, GAGs, lipids, and DNA. Another important advantage of Raman spectroscopy is the low intensity of water bands, which in other spectroscopic methods makes the analysis of biological materials difficult [39]. Raman spectroscopy extracts spatial information from complex biological samples, and as a result, it is an extremely accurate tool for examining a variety of plant materials [46], such as pollen [47,48], fruits [49,50], roots [51], and wood of various origins [52,53,54,55,56,57]. Its mechanism makes it possible to perform analysis of both the molecular composition and molecular structure of cell walls [58,59,60].

**Figure 2:**
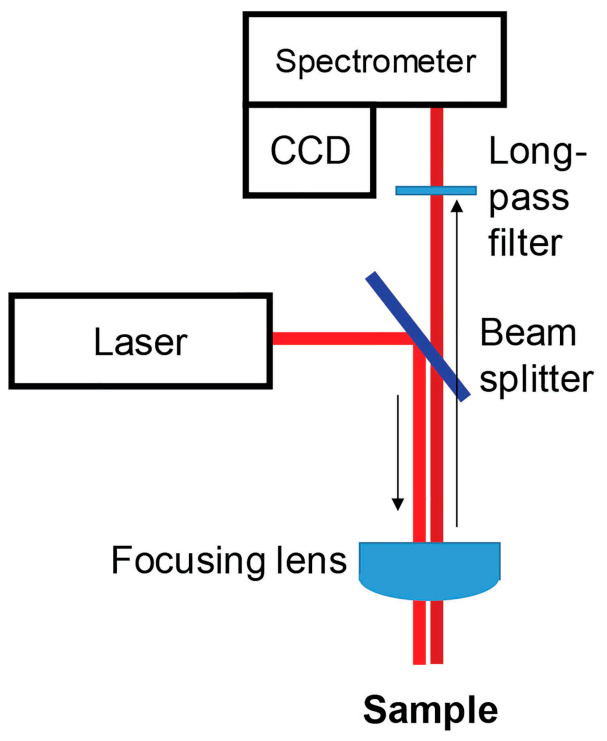
Schematic representation of a Raman spectrometer. Copyright Plant Methods 2021.

A Raman spectrometer consists of a laser source which generates a stream of photons. The light is then directed through a beam splitter and focused through a lens onto the test sample. This leads to scattering of light, which is then usually collected using the same optics and directed into the spectrometer. Elastically scattered photons are cut off by long-pass filters before entering the spectrometer. After the inelastically scattered photons (Raman photons) are scattered on the spectrometer gratings according to their energies, they are captured by the CCD [1,61]. An exemplary diagram of the Raman spectrometer operation is shown in Figure 2 (other settings are possible).

Raman spectroscopy was for a long time used exclusively as a laboratory technique, but there are now several commercially available handheld spectrometers. These instruments typically have laser excitation in the green (λ = 532 nm), red (λ = 785 or 830 nm), or infrared (λ = 1064 nm) parts of the electromagnetic spectrum [22,23,24,62,63,64]. The beam diameter or laser spot size on portable Raman spectrometers ranges from a few dozen microns to a few millimetres. Excitation wavelength is one of the most important parameters to pay attention to in spectroscopic examinations of plants. Results acquired by researchers show that by using radiation in the blue and green parts of the electromagnetic spectrum it is possible, in particular, to visualise carotenoid signals. This results from the strong absorption of carotenoids in this part of the electromagnetic spectrum [65]. Lasers with wavelengths above 561 nm and below 700 nm are not suitable for structural analysis of living plants due to the extremely strong fluorescence of chlorophyll. Chlorophyll fluorescence decreases exponentially at wavelengths above 700 nm. Therefore, laser excitations of 785–830 nm provide sufficient signal spectra for plant leaf noise [66]. Handheld Raman spectrometers provide accurate access to physiological responses in plants under field conditions [66], both for plant producers and researchers [67].

The Raman method is an optical spectroscopic technique which can effectively be used to detect and quantify key compounds without destroying the sample. As a result, it is a powerful tool for monitoring plant physiological status and assessing crop quality, pathology, and plant maturation [67,68,69,70,71,72].

Raman spectroscopy is not yet used widely in agriculture and in response to food analysis but has been used recently [73]. Ramaskie techniques do not require a labor-intensive access service, and they can be tested directly in the environment with pits [74]. The Raman method is fast and non-invasive; there is a text, a spectrometric manual, that prevents spectral registration and fruit testing on the plant or during sorting. An additional advantage of Raman spectroscopy is a small queue for water or its steps, thanks to which you can analyse the attendance and dried fruit. The Raman methodology also enables food safety to be controlled by glass or polymer [75,76]. Raman techniques used in precision agriculture can significantly improve capacities for farming management, crop quality assessment, as well as biological and chemical contaminant detection, thereby contributing to food safety as well as the productivity and profitability of agriculture [77].

## 3. Raman Spectroscopy in Assessment of Changes during Growth as Well as Harvest Maturity of Plants 

Analysis of the spectra collected from various crops at specific vegetation stages can be successfully used to determine the optimal harvest time [78,79]. As an example, Piot and colleagues applied confocal Raman microscopy to examine wheat grain and follow the evolution of protein content and structure during the growth of wheat grains of different cultivars. The cultivars differed in the level of grain hardness and the aptitude to separation of peripheral layers during milling process [80]. The study showed that RS is a suitable tool for acquiring information on grain structure and composition and, most importantly, can detect molecules present at low concentrations, such as α-helical protein. Researchers have noted that concentration of this protein increases as the grains harden during the maturation process [1].

**Figure 3:**
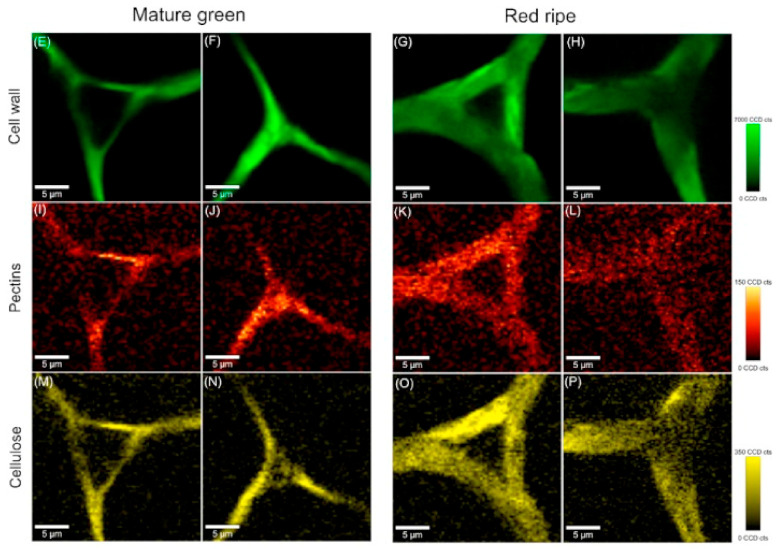
Raman images of cross sections of tomato cell wall from mesocarp at laser with green light (λ = 532 nm); a power of 25 mW and integration time at 0.1 s was chosen. Raman images of all primary cell wall polysaccharides at 2936 cm^−1^, γ(CH) (**E**–**H**). Raman images of pectin at 854 cm^−1^, the (COC) skeletal (**I**–**L**). Raman images of cellulose at 1096 cm^−1^ and 1115 cm^−1^ (glycosidic bond) (**M**–**P**). Copyright Plant Physiology and Biochemistry 2017.

Chylińska and her team applied confocal spectroscopy to assess the ripening stage of tomatoes. Fruit was harvested at the mature green and red ripe stages. The samples were examined using a Raman spectrometer equipped with a green light laser (λ = 532 nm). The researchers investigated the effect of biochemical parameters (such as cell wall polysaccharide content, phenolic compounds, ascorbic acid, and pectinolytic enzyme activity) on cell wall microstructure and changes in polysaccharide distribution during the process of physiological development of tomato fruit (Solanum lycopersicum cv Cerise). The study showed that confocal Raman spectroscopy makes it possible to visualise changes in the spatial distribution of polysaccharides in the plant cell wall (including the central lamella region). In mature green tomato, pectin concentrations were visible particularly in cell corners, whereas ripe red tomato were found with a homogeneous distribution of pectin in the cell wall (Figure 3). The study by Chylińska et al. demonstrates that the Raman technique can visualise the changes occurring in the cell wall (mainly degradation of pectin polysaccharides) during tomato ripening [81].

Lopez-Sanchez et al. [82] used the potential of Raman spectroscopy to assess changes taking place in the process of olive fruit growth and ripening. For this purpose, the researchers measured the spectra of different parts of olive fruit (skin, flesh, and stone) at different stages of development. They observed an increase in carotenoids and phenolic compounds during olive growth and a decrease during the ripening phase. The evolution of the different spectral bands was linked to the content of the olive constituents, such as triglycerides, water, carotenoids, and phenolic compounds. The study demonstrated the ability of RS to track oil accumulation in olive fruit. Increased intensity of the peaks at 1440 cm^−1^ correlates well with oil content in the fruit measured using the standard Soxhlet extraction method. The researchers showed that increase in carotenoid and phenolic contents during olive growth and decrease in these values during the ripening stage can effectively be monitored using vibration bands at 1525 and 1605 cm^−1^.

Szymańska-Chargot et al. [83] applied confocal Raman microscopy to assess changes in the distribution of polysaccharides in the cell wall of apple flesh in ripening fruit and during storage after harvest. The apples were collected on three dates: at 1 month and at 2 weeks before the optimum harvest date and on the optimum harvest date. Apples collected on the optimum harvest date were kept in storage for 3 months. The researchers acquired Raman maps for each harvest date and after 1, 2, and 3 months of storage. Raman images of apple cell walls show significant changes in the quantity and distribution of the main cell wall polysaccharides. Analysis of the Raman maps showed degradation of pectins distributed in the middle lamella and primary cell wall. These findings were consistent with the results of chemical assays. During the process of apple ripening and ageing, the analyses based on Raman spectroscopy showed changes in the distribution of pectins, which in young fruit were dispersed along the cell walls, whereas in ripe fruit and those kept in storage they were concentrated in the cell wall corners. Analysis of apples after 3 months in storage showed a significant decrease in pectin content. The findings reported by this research team show that Raman imaging can be a very useful tool for early identification of changes in plant tissue composition during development.

Qin et al. [84] report that it is possible to use Raman spectroscopy to visualise the content of lycopene, the main carotenoid in tomatoes. According to those researchers, imaging of changes in lycopene content during fruit ripening is a good method for monitoring the stage of tomato maturity. During the study, the research team developed a laboratory-based point-source Raman chemical imaging system to detect and visualise the internal distribution of lycopene during the process of fruit ripening and post-harvest. The researchers analysed lycopene content in tomato fruit samples representing different stages of ripeness (i.e., green, breaker, turning, pink, light red, and red). Qin et al. applied the spatially offset Raman spectroscopy (SORS) technique for subsurface detection of a Teflon slab placed under samples of the outer pericarp from green and pink tomatoes. The findings showed that the Teflon spectrum acquired this way can be extracted from SORS measurements of tomato pericarp placed above Teflon. These results suggest a potential for the development of the SORS method in imaging lycopene concentration as a marker of tomato fruit ripeness.

Martin et al. [85] developed a tomato ripening model based on vibrational bands of carotenoids in Raman spectra. Tomato fruit during the growth phase and during the post-harvest ripening stage were analysed using a laboratory Raman spectrometer equipped with a 532 nm laser. The researchers observed increase in the carotenoid signal at the start of the turning stage of the fruit ripening. The acquired data were used by the team to build a model describing the stages of the tomato ripening process and helping to accurately assess post-harvest fruit quality.

**Figure 4:**
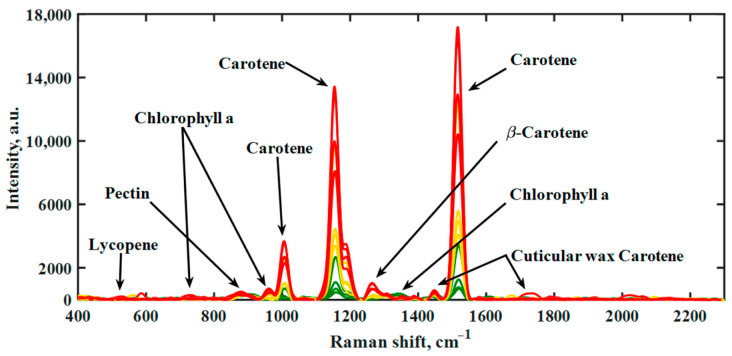
Raman spectra of hot peppers during the maturation process obtained with handheld Raman spectrometer at 785 nm laser wavelength with a power of 100 mW. Unripe pepper (green), ripening pepper (yellow), and fully ripe fruit (red) are each represented by four deliberately selected spectra. Copyright MDPI 2021.

A similar study using handheld Raman spectrometers for hot pepper was first carried out by Langer et al. [86] Scientists in their reports described the Raman signals of carotenoids typical of hot pepper fruits and followed their evolution during maturation (Figure 4). Researchers proposed and compared a multivariate chemometric model and a simple one-dimensional model, resulting in a four-point scale for grading the ripeness of hot pepper fruit. The authors suggest that the model proposed is appropriate for assessing the ripening stage of fruit containing carotenoid, and thus for determining the maturity on site or during the sorting process in an automated manner.

Bands in the range of approximately 800 to 1600 cm^−1^ have been assigned to carotenoids. The band at about 860 cm ^−1^ can be attributed to the asymmetric stretching of the C–O–C glycosidic bond in acid pectins. A faint band at 1327 cm^−1^ is attributed to chlorophyll a, which is known to have the highest intensities in the pure chlorophyll spectrum. The most intense bands in the spectrum were assigned to the carotenoids, observed at 1150–1170 and 1500–1550 cm^−1^. The band is formed as a result of vibrations in the C = C phase and C–C stretching of the polyene chain. In the range of 1000–1020 cm^−1^, methyl groups attached to the polyene chain were recognized, showing moderate intensity [86].

Cabrales and team applied CRM to examine cross-sections of growing cotton fibres during the main five developmental stages. In course of the study, the researchers analysed vibrational bands at 383 cm^−1^, attributed to cellulose. The intensity of the Raman spectra for cellulose was significantly lower for fibres harvested at 21 days post-anthesis (dpa) compared to 56 dpa. The sub-micron resolution of the CRM provides insight into the deposition of cellulose in the secondary cell wall. The findings showed that it is possible to obtain information on the chemical composition and structure of deposited cellulose in developing cotton fibres. CRM can be used as a tool to differentiate between cotton fibres at different stages of growth [87].

## 4. Raman Spectroscopy for Fruit and Seeds Quality Control

RS can also be applied to perform non-invasive assessment of the nutritional value and quality of plants, fruits, and seeds, and as a result, it is a perfect tool to be used in digital agronomy [88,89]. Nekvapil et al. [41] investigated the relationship between the freshness of selected citrus fruit varieties and their Raman spectra. Citrus fruit freshness is associated with the appearance and colour and consequently the net carotenoid content of the peel. In that study the research team assessed freshness of commercially available citrus fruits (clementines and different varieties of mandarins) using a handheld Raman spectrometer. Evaluation of fruit freshness can be performed using Raman instruments quickly, objectively, and without destroying the material. The samples were excited with 532 nm and 785 nm waves. Nekvapil et al. evaluated the fruit for carotenoid contents in the peel during the time span between fresh fruit delivery and their physical degradation. The analyses found a strong correlation between carotenoid content of the peel and the intensity of the Raman signal. The findings showed that the intensity of the Raman signal for carotenoids is a good indicator of fruit freshness. Based on this, the researchers introduced a Raman coefficient of freshness (CFresh), which decreases linearly in time with a different slope for different citrus groups. After 7 days of the experiment, a decrease in signal intensity was observed in the case of all the samples, but this change was varied. It was observed that the decrease in Raman signal intensity was smaller in the case of fruit stored in daylight compared to fruit kept in dark storage. The Raman coefficient of freshness (CFresh) calculated for specific fruit appears to be a user-friendly, fast, and sensitive method for assessing citrus fruit freshness with a portable Raman spectrometer. The portable system shows great potential for extensive use in the evaluation of citrus freshness, both in ripening crops and in fruit supplied to the market or to the food industry.

Similarly, Nikbakht and colleagues [90] applied RS to determine tomato fruit quality. The researchers demonstrated that RS can be used to measure important tomato quality parameters such as soluble solids content (SSC), acidity (pH), and colour. They showed that RS can be very effective in assessing the quality of both the external and internal properties of tomatoes.

Zhu et al. [89] applied Raman spectroscopy to examine the mechanisms underlying fruit lignification. Knowledge of the fruit lignification process would facilitate operations aimed to optimise storage and preservation strategies and to reduce post-harvest deterioration. By investigating lignin deposition in fruit at cellular level it may be possible to work out the mechanisms underlying fruit lignification. The study mainly aimed to establish a procedure for applying Raman microspectroscopy to visualise cellular-level lignification in loquat fruit. Fruit lignification leads to increased fruit firmness and is important from the viewpoint of optimum post-harvest handling of fruit to minimise deterioration. The findings of the study showed that Raman spectroscopy can effectively be used to assess fruit lignification in order to determine fruit maturity.

The group of Morey and Kurouski et al. [91] used RS to assess nutrient content of potato tubers. The researchers showed that the intensity of the 479 cm^−1^ band (correlating with starch) increases linearly with growing starch content in the test samples. These findings suggest that RS can effectively be used to measure starch content in intact potato tubers. Using such calibration curves, the researchers were able to accurately determine the absolute starch concentration in potatoes. The findings showed differences in the spectra collected from samples with different starch contents; the Raman spectra acquired from a sample containing 12% starch (6 g starch) were statistically different from the spectra collected from samples with starch at a content of 9% (4.5 g starch) and 15% (7.5 g starch). Similarly, the spectra acquired from samples with 15% starch content differed significantly from the spectra obtained from samples with 12 and 18% starch content. The results of the analyses and the standard deviations obtained suggest that this method can be used to identify starch content within 3% accuracy. It can be expected that more careful standardisation may change the prediction accuracy to 1% and below.

Krimmer et al. [92] used a Raman spectrometer to assess the nutrient content of maize grains. Maize is popular worldwide as an ingredient in human food and livestock feed as well as a raw material in industry and a biofuel. The researchers found that Raman spectroscopy can identify carbohydrates, fibre, carotenoids, and proteins in maize kernels [63]. Abreu and colleagues applied RS to monitor coffee quality. They collected spectra from coffee beans stored under different conditions for 0, 3, 6, 9, 12, and 18 months. The researchers observed that changes in kahweol visualised by means of vibrational bands can be helpful in predicting coffee quality and changes taking place in beans kept in storage.

## 5. Raman Spectroscopy for Determination of Species and Origin of Plants, Fruit, and Seeds

Related publications have reported successful attempts to build RS-based models enabling identification of fruit varieties. Feng and colleagues applied RS to study eight different citrus fruits. The team succeeded in building a model to distinguish between citrus varieties. This work shows that RS can be used to accurately, quickly, and efficiently identify varieties and assess citrus fruit quality [93].

Kurouski et al. [91] focused on using SR to distinguish potato varieties. Owing to their high starch content, simple cultivation process, and high yield, potatoes are one of the basic ingredients in the diet of people across the world. Potato tubers consist of approximately 83% water and 12% carbohydrates, whereas proteins, vitamins, and other trace elements account for the remaining 4%. The precise composition of potatoes varies relative to the type of potato and the place where they are grown. The researchers successfully used Raman spectroscopy to identify nine different potato varieties and to determine the origin of the crop. Using spatially offset Raman spectroscopy (SORS), the researchers observed that the peak intensity varied between potato varieties at 479 and 1125 cm^−1^ for starch, 1600 and 1630 cm^−1^ for phenylpropanoid, 1527 cm^−1^ for carotenoid content, and 1660 cm^−1^ for protein content. Based on the data obtained, Kurouski’s team was able to identify the potato variety and determine the location of the potato crop with an accuracy of 81 to 100%.

Farber and colleagues [18] showed that by acquiring Raman spectroscopy spectra of peanut leaves it is possible to identify different peanut varieties and genotypes. This method can also be applied to determine plant resistance to nematodes and to measure the oleic/linoleic oil (O/L) ratio. Furthermore, analysis of peanut seeds based on Raman spectroscopy can be applied to accurately identify the genotype as well as carbohydrates, proteins, fibre, and other nutrients. In course of their experiment, the researchers grew plants of 10 different peanut genotypes and analysed their spectra. All the genotypes were found with similar profiles, with vibrational bands characteristic of carbohydrates, cellulose, pectins, carotenoids, phenylpropanoids, protein, and carboxylic acid. The findings acquired using the specially developed PLS-DA model showed that the Raman method makes it possible to identify peanut varieties with 80% accuracy. The results of the study suggest that the resistance of peanut plants to nematodes is related to changes in carotenoid- and phenylpropanoid-specific peaks. In the food industry, peanuts with a high oleic ratio are preferred because of their longer shelf life and consequently reduced rancidity. It has also been shown that high-oleic peanuts beneficially affect (i.e., decrease) serum cholesterol levels and reduce the risk of cardiovascular disease. RS showed that plants of this specific genotype had lower phenylpropanoid contents, while all other peaks remained almost identical. Farber and the team reported 82% accuracy of the Raman method in identifying peanuts with high versus normal oleic ratios. To compare the accuracy of the method, the researchers performed Raman analyses of peanut seeds. The findings show 95% accuracy of Raman spectroscopy in identification of peanut seeds, compared to 82% in the case of leaves.

Krimmer et al. [63] investigated the feasibility of Raman spectroscopy in identification of maize varieties. Using PLS-DA, Krimmer and colleagues showed that RS can be used to identify six different maize varieties based on their unique spectra. In the course of the experiment, Krimmer and colleagues collected over 600 spectra from six different maize varieties. All six varieties had similar spectral profiles, except for the scanned darker kernels, which had a lower intensity. This is due to the different light absorption and scattering properties of these maize kernels, which affect the scanning. This problem can be solved by normalisation, especially at the 1458 cm^−1^ peak displayed by all spectra display. The authors analysed the intensity of the bands at 479 cm^−1^ (starch), 1530 cm^−1^ (carotenoids), 1600/1632 cm^−1^ (both fibres), and 1640–1670 cm^−1^ (protein region) to quantify the carbohydrate, carotenoid, fibre, and protein content of maize. Krimmer and colleagues showed that RS in combination with chemometric methods can be used for highly accurate typing of maize varieties.

Abreu and colleagues in their study showed that RS can be used to discriminate Arabica coffee genotypes very accurately. [92]. Using Raman spectroscopy and principal component analysis, Figueiredo and co-workers were able to identify four Arabica coffee genotypes: one Mundo Novo line and three Bourbon lines, with an accuracy of ~ 80% [94]. Keidel and co-workers [95] showed that spectroscopic measurements of both ground and whole beans can be used to predict the geographical origin of coffee beans. 

## 6. Conclusions 

Raman spectroscopy has been used in analysis of plant biomass for nearly 30 years [96,97]. In plant science, the Raman spectroscope was first used by Atella and Agarwal in the 1980s [52,96,97]. Owing to recent technological advancements, today Raman spectroscopy and the related instruments are recognised as precise tools for studying plant tissues [97,98]. Currently, there are over 25 different types of Raman spectroscopy techniques. For example, a Fourier transformation (FT) Raman spectrometer using a near-infrared (NIR) laser solves the problem of fluorescence interference [99]. The surface-enhanced Raman spectroscopy technique enhances the Raman scattering signal [100]. Raman confocal microscopy provides three-dimensional images of the structure and composition of the material with micrometric resolution and clear image quality [101]. Coherent anti-Stokes Raman scattering–(CARS) provides spectral information with excellent sensitivity and low laser power [102]. Raman resonance scattering (RRS) allows the study of a spectrum of materials in the range of the photon energy itself [103]. Raman optical activity (ROA) monitors a small difference in the scattering of right- and left-circularly polarized light. ROA spectra are sensitive to chirality (e.g., to the enantiomeric excess) and fine variations in molecular geometry including conformational states [104].

Raman microscopy is widely used to examine the composition of the plant cell wall [105]. Numerous studies have reported that Raman imaging techniques can successfully be applied to investigate differences between tissues and to monitor changes in plant cells [35,80,82,106,107,108,109,110,111,112]. Raman spectroscopy can be used to acquire marker-free spectral maps of biological tissues and cells for the needs of chemical, structural, and environmental analyses. Raman images present information about molecular structure, composition, and interactions in micrometres or even nanometres [54,55]. An important advantage presented by the Raman techniques is the fact that Raman signal processing can be used to obtain analysis of several samples or plant fragments of one type [60].

Agriculture and plant growing, as well as plant pathology, are relatively new areas in RS-based research. The related literature published in the recent years shows that Raman spectroscopy can successfully be used in diagnostics of biotic as well as abiotic stresses in plants. Rapid assessment of plant phenotype allows farmers to intervene immediately and precisely to mitigate biotic and abiotic stresses [11,17,22,23,24,26]. Since they are highly sensitive to small changes in plant biochemistry, Raman spectrometers make it possible to identify plant species and plant varieties and allow farmers to select and grow plants with advantageous genotypes [18,63].

This review shows the potential of RS for the digital agriculture of the future. Portable and rapid Raman spectrometry analysis allows for quick detection of biotic and abiotic stresses in plants. Moreover, Raman techniques can be used as an advanced method for plant breeding and selection as they are both non-invasive and non-destructive. RS can also be used for plant phenotyping and nutrient analysis. Advantages presented by RS will certainly become more obvious to others, and the use of Raman spectrometry in digital agriculture will become more widespread. The relatively high cost of the related equipment is a significant drawback for farmers, adversely affecting widespread use of Raman spectroscopy in crop monitoring. It can be expected that continued advancements in the technology will bring the cost of these devices down in the near future. Furthermore, application of Raman spectrometry in agriculture is likely to be implemented in the near future as a service providing farmers with information on the condition of the field along with GPS coordinates of the assessed locations.

The above review takes a closer look at the potential of Raman spectrometers to be used for the advancement of digital agriculture. The related gains outlined here are gradually becoming more obvious for farmers and investors, and the use of Raman spectroscopy in digital agriculture will become more widespread [8].

## Figures and Tables

**Table 1:** Assignment of bands in the Raman spectra of cell wall polysaccharides based on the literature [8]. Copyright Front. Plant Sci. 2021.

Band (cm^−1^)	Vibration Mode	Assigment
480	C–C–O and C–C–C Deformations; Related to glycosidic ring skeletal deformations δ(C–C–C) + τ(C–O) Scissoring of C–C–C and out·of·plane bending of C–O	Carbohydrates
520	ν(C–O–C) Glycosidic	Cellulose
747	γ(C–O–H) of COOH	Pectin
849–853	(C_6_–C_5_–O_5_–C_1_–O_1_)	Pectin
917	ν(C–O–C) In plane, symmetric	Cellulose and phenylpropanoids
964–969	δ(CH_2)_	Aliphatics
1000–1005	In plane CH_3_ rocking of polyene aromatic ring of phenylalanine	Carotenoids and protein
1048	ν(C–O) + ν(C–C) + δ(C–O–H)	Cellulose and phenylpropanoids
1080	ν(C–O) + ν(C–C) + δ(C–O–H)	Carbohydrates
1115–1119	Sym ν(C–O–C); C–O–H bending	Cellulose
1155	C–C Stretching; ν(C–O–C), ν(C–C) in glycosidic linkages, asymmetric ring breathing	Carotenoids and carbohydrates
1185	ν(C–O–H) Next to aromatic ring + δ(CH)	Carotenoids
1218	δ(C–C–H)	Carotenoids, xylan
1265	Guaiacyl ring breathing, C–O stretching (aromatic); –C=C–	Phenylpropanoids, unsaturated fatty acids
1286	δ(C–C–H)	Aliphatics
1301	δ(C–C–H) + δ(O–C–H) + δ(C–O–H)	Carbohydrates
1327	δCH_2_ Bending	Aliphatics, cellulose, and phenylpropanoids
1339	ν(C–O); δ(C–O–H)	Carbohydrates
1387	δCH_2_ Bending	Aliphatics
1443–1446	δ(CH_2_) + δ(CH_3_)	Aliphatics
1515–1535	–C=C– (in plane)	Carotenoids
1606–1632	ν(C–C) Aromatic ring + δ(CH)	Phenylpropanoids
1654–1660	–C=C–, C=O Stretching, amide I	Unsaturated fatty acids
1682	COOH	Carboxylic acids
1748	C=O Stretching	Esters, aldehydes, carboxylic acids and ketones

## Data Availability

Not applicable.

## Abbreviations

CARS	Coherent anti-Stokes Raman scattering
CRM	Confocal Raman spectroscopy
DNA	Deoxyribonucleic acid
FT	Fourier transformation
GAGs	Glycosaminoglycans
NIR	Raman near-infrared
ROA	Raman optical activity
RS	Raman spectroscopy
RRS	Raman resonance scattering
SORS	Spatially offset Raman spectroscopy
PLS-DA	Partial least-squares discriminant analysis
